# MTHFR C677T Polymorphism and Risk of Congenital Heart Defects: Evidence from 29 Case-Control and TDT Studies

**DOI:** 10.1371/journal.pone.0058041

**Published:** 2013-03-11

**Authors:** Wei Wang, Yujia Wang, Fangqi Gong, Weihua Zhu, Songling Fu

**Affiliations:** 1 Children’s Hospital, Zhejiang University School of Medicine, Hangzhou, P. R. China; 2 Centre Hospitalier de l’Université de Montréal, CRCHUM-Hôpital Notre-Dame, Montreal, Quebec, Canada; MOE Key Laboratory of Environment and Health, School of Public Health, Tongji Medical College, Huazhong University of Science and Technology, China

## Abstract

**Background:**

Methylenetetrahydrofolate reductase (MTHFR) is an important enzyme for folate metabolism in humans; it is encoded by the *MTHFR* gene. Several studies have assessed the association between *MTHFR* C677T polymorphism and the risk of congenital heart defects (CHDs), while the results were inconsistent.

**Methods and Findings:**

Multiple electronic databases were searched to identify relevant studies published up to July 22, 2012. Data from case-control and TDT studies were integrated in an allelic model using the Catmap and Metafor software. Twenty-nine publications were included in this meta-analysis. The overall meta-analysis showed significant association between *MTHFR* C677T polymorphism and CHDs risk in children with heterogeneity (*P*_heterogeneity_ = 0.000) and publication bias (*P*_egger_ = 0.039), but it turned into null after the trim-and-fill method was implemented (OR = 1.12, 95% CI = 0.95–1.31). Nevertheless, positive results were obtained after stratified by ethnicity and sample size in all subgroups except the mixed population. For mothers, there was significant association between the variant and CHDs without heterogeneity (*P*_heterogeneity_ = 0.150, OR = 1.16, 95% CI = 1.05–1.29) and publication bias (*P*_egger_ = 0.981). However, the results varied across each subgroup in the stratified analysis of ethnicity and sample size.

**Conclusions:**

Both infant and maternal *MTHFR* C677T polymorphisms may contribute to the risk of CHDs.

## Introduction

Congenital heart defects (CHDs) refer to the defects in the structure of the heart and great vessels present at birth. The incidence of CHDs is reported to be 5–8‰, making them one of the most common congenital disorders in newborns. Besides, they are the leading causes of infant death worldwide, with a mortality of 24.1% [1]. Yet, the etiology of CHDs is not fully understood. CHDs like other multifactorial disease are proved to be affected by both genetic and environmental factors [2]. Recently, some studies have identified the association between the lack of folate (also called folic acid or vitamin B9) and CHDs risk. The deficiency in folic acid is known to result in hyperhomocysteinemia, which is one of the proved risk factors related to the occurrence of CHDs [3].

Methylenetetrahydrofolate reductase (MTHFR), as a crucial enzyme for the metabolism of folate, plays an important role in processing amino acids and building blocks of proteins. Specifically, it converts 5,10-methylenetetrahydrofolate to 5-methyltetrahydrofolate. This reaction is a multistep process, in which, MTHFR converts the homocysteine to another amino acid, methionine. Then, the methionine was used by body to make proteins and other important compounds [4]. *MTHFR* gene is located at 1p36.3 of humans. The missense mutation, C677T (rs1801133), is one of the most investigated single nucleotide polymorphisms (SNPs) [5]. It has reported that the SNP produces a thermolabile variant of the *MTHFR* with reduced enzymatic action, which causes increased plasma homocysteine concentrations [6]. The homozygous 677TT and heterozygotes 677CT genotypes have approximately 30% and 65% of the enzyme activity of MTHFR respectively compared with the 677CC genotype [7]. The homozygous 677TT genotype leads to mildly elevated plasma homocysteine and lower folate levels, while either the 677CT or 677CC genotype brings about higher plasma homocysteine and folate levels [8].

Junker and colleagues firstly proposed that infant *MTHFR* 677TT genotype was a risk factor for CHDs in a case-control study of 114 Causasian patients and 228 healthy controls. After this report, a string of studies have subsequently explored the association between this variant and risk of CHDs, however the results were controversial. Besides, single study may have limited statistical power to detect the modest effect of *MTHFR* C677T polymorphism on CHDs. Therefore, we conducted a meta-analysis of the available data and used the method described by Kazeem et al. [9] to integrate the results from case-control and transmission/disequilibrium test (TDT) studies to provide statistical powerful evidence on the role of *MTHFR* C677T polymorphism in CHDs.

## Materials and Methods

We designed and reported the meta-analysis according to the Preferred Reporting Items for Systematic Reviews and Meta-analyses (PRISMA) statement (Checklist S1).

### Identification and Eligibility of Relevant Studies

We conducted an electronic search for relevant articles published before July 22, 2012 in PubMed, EMBASE, and ISI Web of Science databases with the combination of the following terms: “congenital cardiac/heart defects”, “methylenetetrahydrofolate reductase or *MTHFR*” and “polymorphism or variant”. To expand the coverage of our searches, we further carried out searches in Chinese National Knowledge Infrastructure (CNKI) and Wanfang databases with the translation of all English searching terms. Reference lists of the retrieved articles were also scanned for more eligible studies. We included case-control studies and TDT with human subjects that investigated the association between *MTHFR* C677T polymorphism and CHDs risk in all languages. All phenotypes of CHDs, such as patent formen ovale, atrial septal defect, patent ductus arteriosus, coarctation of the aorta, were included in this meta-analysis. Animal studies, reviews, simply commentaries, case reports and unpublished reports were excluded. Additionally, studies with overlapping data were carefully examined, and the study that included the largest number of subjects was finally selected.

### Data Extraction

All data were extracted independently by two authors (W. Wang and YJ. Wang), and any disagreement was adjudicated by the corresponding author. The following information was extracted or calculated from each study: first author, year of publication, country of origin, ethnicity, type of CHDs, study design, number of cases and controls, counts of alleles in case and control groups in case-control studies and numbers of transmitted alleles from heterozygous parents to affected offspring in family-based studies. If there were multiple ethnic populations included in a study, each population was considered separately.

### Statistical Analysis

Hardy-Weinbery equilibrium (HWE) in controls was assessed again in each included studies by the goodness-of-fit *χ^2^* test. Data from case-control and TDT studies were summarized in two-by-two and two-by-one tables respectively. Odds ratios (ORs) and their 95% confidence intervals (CIs) were recalculated based on allele data using the method reported by Kazeem et al. [9]. Heterogeneity was detected by the *χ^2^*-based Cochran’ *Q*-test, in which heterogeneity was considered significant at *P*<0.1. Pooled OR was calculated by the fixed-effect model when heterogeneity was negligible, whereas, the random-effect model was adopted. An allelic model was assumed for *MTHFR* C677T to combine results of case-control or TDT studies. For the synthesis of case-control and TDT studies, the method described by Kazeem et al. [9] was used. If a study used case-control and TDT designs in the overlapping probands, the design with less sample size was excluded. Overall meta-analysis was initially performed. Then we conducted stratification analyses if data permitted, according to ethnicity (Asian, European and mixed populations), sample size (larger-sample-size subgroup: number of cases in case-control study or number of family in family-based study ≥100; small-sample-size subgroup: number of cases in case-control study or number of family in family-based study <100) and study design (case-control and TDT studies). In addition, sensitivity analysis was conducted to assess the influence of each individual study on overall estimate by sequential removal of individual studies. Publication bias was assessed using funnel plots and Egger’s test. If there was some evidence of publication bias, the trim and fill method which estimates the number and results of potential missing studies resulting from publication bias was applied. All the *P* values were two-sided with a significant level at 0.05 except for the one in *Q* test for heterogeneity, and all analyses were carried out with Catmap and Metafor software in R program (version 2.9.2).

## Results

### Characteristics of Including Studies

**Figure 1:**
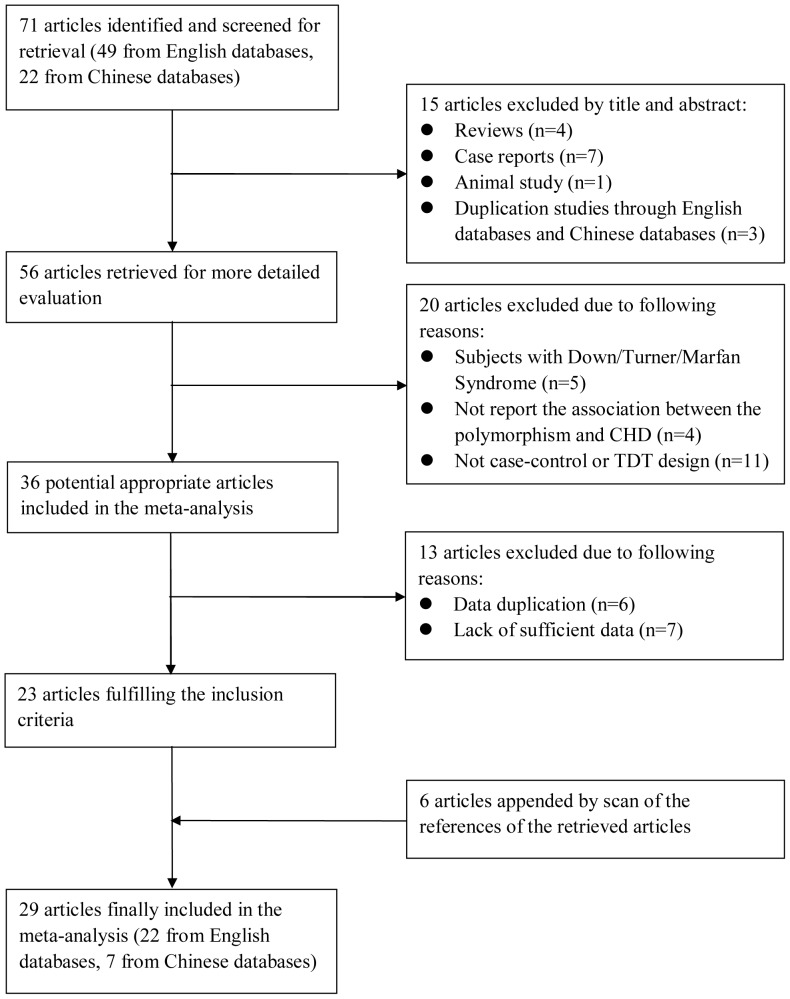
Flow diagram of the study selection procedure.

Figure 1 shows the literature search and study selection procedures. Our literature search identified 71 publications, of which 15 were excluded after review of title and abstract. Thirty-three publications were further excluded after review of full-text because of the following reasons: subjects with Down/Turner/Marfan Syndrome (5 studies), no report of the association between *MTHFR* C677T polymorphism and CHDs (4 studies), without case-control or TDT design (11 studies), data duplication (6 studies) and lack of sufficient data (7 studies). Nevertheless, 6 publications were appended by scan of the references of the retrieved articles. Thus, 29 publications involving 2554 cases and 3838 controls in 20 case-control studies for children, 1837 cases and 16351 controls in 14 case-control studies for mothers, and 572 families in 2 TDT studies were ultimately included [2], [10]–[37]. Of these 29 studies, 13 were conducted in Asians, 7 in Europeans, and 9 in mixed or unknown ethnicity. Besides, 12 and 9 studies involved all types of CHDs or specific types of CHDs separately (including contruncal, congenital atrial septal defect, patent ductus arteriosus, tetralogy of fallot), and the rest of studies were lack of related information. Except for 7 studies, the distribution of genotypes in the controls was consistent with HWE. Table 1 shows the characteristics of the included studies.

**Table 1:** Characteristics of the included studies.

First author	Publication year	Country	Ethnicity	Types of CHD	Study design	Subject	Case/control (family)	HWE
Junker	2001	Germany	European	All types except PFO	CC	Child	114/228	Yes
Wenstrom	2001	USA	Black27%, White69%, others4%	All types	CC	Child	26/116	No
Storti	2003	Italy	European	Conotruncal heart defects	CC	Child, mother	103/200, 103/200	Yes
Yan-a	2003	China	Asian	–	CC	Child	187/103	-
Yan-b	2003	China	Asian	–	CC	Mother	187/102	Yes
McBride	2004	USA	Mixed	All types	TDT	-	197	Yes
Nurk	2004	Norway	–	–	CC	Mother	25/14484	Yes
Shaw	2005	USA	White non-Hispanic 67%, White Hispanic 23%, other 10%	Conotruncal heart defects	CC	Child	153/434	Yes
Lee	2005	China	Asian	All types	CC	Child	213/195	Yes
Liu	2005	China	Asian	Conotruncal heart defects	CC	Child	97/118	Yes
Li	2005	China	Asian	All types	CC	Child, mother	183/103,183/102	Yes
Zhu	2006	China	Asian	ASD, PDA	CC	Child, mother	56/103, 56/102	Yes
Van Beynum	2006	Netherlands	European	All types	CC	Child, mother	165/220, 158/261	Yes
Hobbs	2006	USA	32.4% for whites, 12.5% for African-Americans, and 40.9% for Hispanics	Septal, conotruncal, right-left sided CHD	TDT	-	375	Yes
Zhong	2006	China	Asian	All types	CC	Mother	115/115	–
Liu	2007	China	Asian	–	CC	Child	132/107	–
Galdieri	2007	Brazil	White22%, non-White 78%	–	CC	Child, mother	58/38, 47/26	Yes
Wintner	2007	Austria	European	All types	CC	Mother	31/31	Yes
Van Driel	2008	Netherlands	European	All types	CC	Child, mother	229/251, 230/251	Yes
Li	2009	China	Asian	–	CC	Child	104/208	Yes
Gong	2009	China	Asian	–	CC	Child	76/76	–
Marinho	2009	Portugal	European	Tetralogy of fallot	CC	Child	38/251	No
Hu	2009	China	Asian	–	CC	Child	36/40	–
Peng	2009	China	Asian	All types	CC	Mother	91/101	Yes
Kuehl	2010	USA	European	CoAo	CC	Child	55/300	Yes
Xu	2010	China	Asian	All types	CC	Child	502/527	Yes
García-Fragoso	2010	Puerto Rico	–	All types	CC	Child, mother	27/220, 27/220	Yes
Hobbs	2010	USA	White 78.1%, African American 15.2, Hispanic 5.2%, others 1.4%	Septal, conotruncal, right-left sided CHD	CC	Mother	553/356	Yes
Balderrábano-Saucedo	2012	Mexico	Mixed	All types	CC	Mother	31/62	Yes

Abbreviations: CC, case-control study; TDT, transmission/disequilibrium test; CHD, congenital heart defect; PFO, patent formen ovale; ASD, atrial septal defect; PDA, patent ductus arteriosus; CoAo, coarctation of the aorta; HWE, Hardy-Weinbery equilibrium.

### The Association between *MTHFR* C677T Polymorphism and CHDs Risk in Children

**Figure 2:**
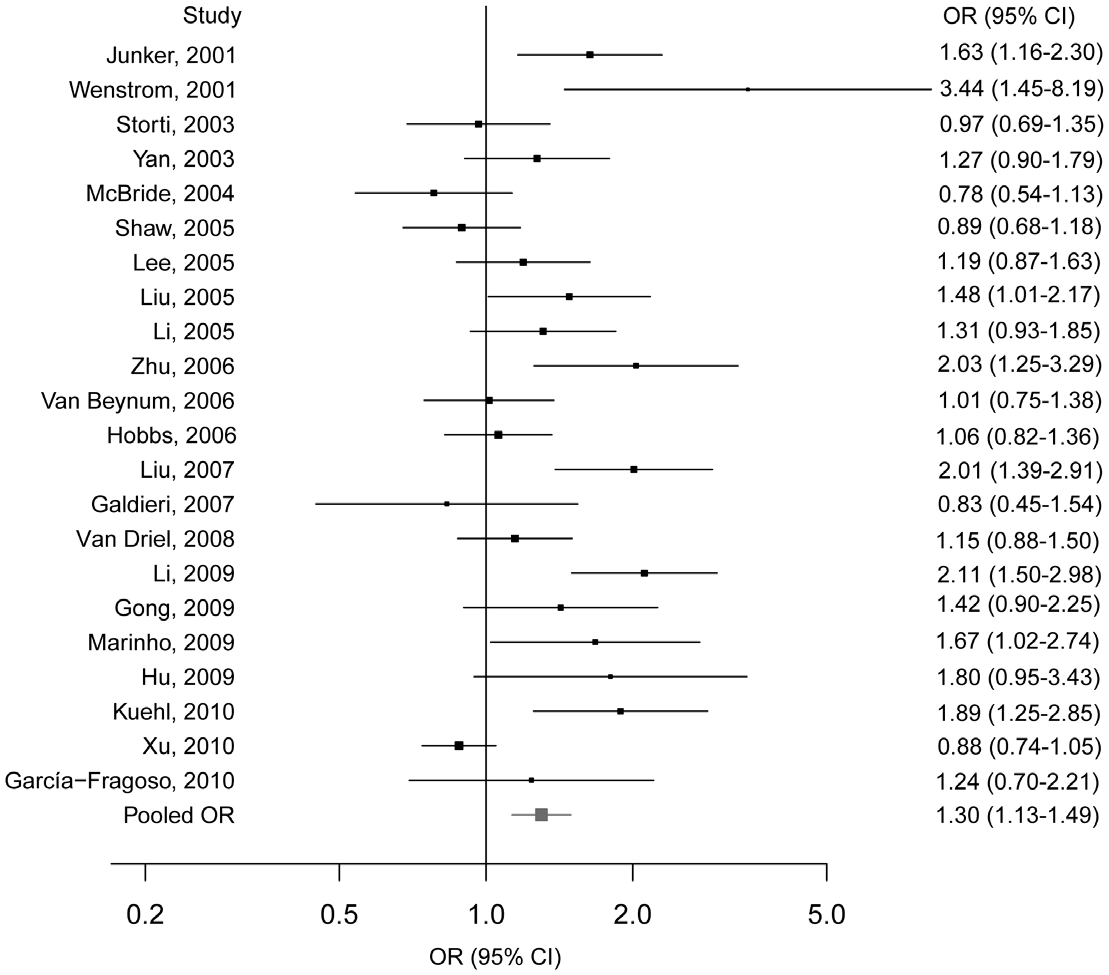
The forest plots of ln(OR) with 95%CIs for the *MTHFR* C667T in children for CHDs. Random-effects pooled OR = 1.30, 95% CI = 1.13–1.49, *P* = 0.000; *χ* ^2^ = 13.65, *P*_heterogeneity_ = 0.000.

Among the 22 studies of the *MTHFR* C677T polymorphism in children, significant heterogeneity was detected (*P*_heterogeneity_ = 0.000), thus the random-effects model was employed. Compared to the C allele, the T allele conferred a pooled OR of 1.30 (95% CI = 1.13–1.49) in allelic model (Figure 2). Because of significant heterogeneity for the overall meta-analysis observed, a sensitivity analysis was conducted in an attempt to assess the effect of each study on the pooled estimate. The result showed the pooled ORs for the allelic model were similar before and after elimination of each study (Table S1). As reflected in funnel plot (Figure S1) and Egger’s test, there was significant publication bias in overall meta-analysis (*P*_egger_ = 0.039), therefore, the trim-and-fill method under random-effects model was implemented. However, no significant association between *MTHFR* C677T polymorphism and CHDs risk (OR = 1.12, 95% CI = 0.95–1.31, *P = *0.188) was found after 6 virtual studies were appended.

To investigate the potential source of heterogeneity, stratified analysis was initially performed by ethnicity (Table 2). After stratified by ethnicity, significant between-heterogeneity was not materially altered (*P*_heterogeneity_ in all subgroups < 0.10). In the Asian or European population, the allelic model exhibited significant association with CHDs risk (Asian: OR = 1.46, 95% CI = 1.16–1.83; European: OR = 1.30, 95% CI = 1.04–1.62), whereas there was no positive reslut in the mixed population (OR = 1.01, 95% CI = 0.76–1.35). Then the data were further stratified by sample size into larger-sample-size and small-sample-size subgroups (Table 2). Significant heterogeneity was detected in large-sample-size subgroup (*P*_heterogeneity_ = 0.000), but not in small-sample-size subgroup (*P*_heterogeneity_ = 0.250). Nevertheless, significant associations between *MTHFR* C677T polymorphism and the risk of CHDs were observed in both subgroups (large-sample-size subgroup: OR = 1.18, 95% CI = 1.01–1.38; small-sample-size subgroup: OR = 1.60, 95% CI = 1.32–1.94). The stratified analysis by study design could not be performed due to only 2 TDT studies obtained.

**Table 2:** Stratified analyses of the *MTHFR* C667T polymorphism in association with CHD risk under allelic model.

Variables	Case/control (famliy)	OR (95% CI)	*P*	*P*_heterogeneity_
Child				
Ethnicity				
European(n = 6)	704/1450	1.30 (1.04–1.62)	0.020	0.034
Asian (n = 10)	1586/1580	1.46 (1.16–1.83)	0.001	0.000
Mixed (n = 5)	237/588 (572)	1.01 (0.76–1.35)	0.946	0.031
Sample size				
Large (n = 13)	2085/2576 (572)	1.18 (1.01–1.38)	0.043	0.000
Small (n = 9)	469/1262	1.60 (1.32–1.94)	0.000	0.250
Mother				
Ethnicity				
European(n = 4)	522/743	1.03 (0.87–1.22)	0.701	0.334
Asian (n = 5)	632/522	1.36 (1.14–1.62)	0.001	0.750
Mixed (n = 3)	631/444	1.25 (0.72–2.19)	0.429	0.028
Sample size				
Large (n = 7)	1529/1387	1.14 (1.02–1.27)	0.024	0.644
Small (n = 7)	308/15026	1.21 (0.87–1.68)	0.248	0.040

### The Association between *MTHFR* C677T Polymorphism and CHDs Risk in Mothers

**Figure 3:**
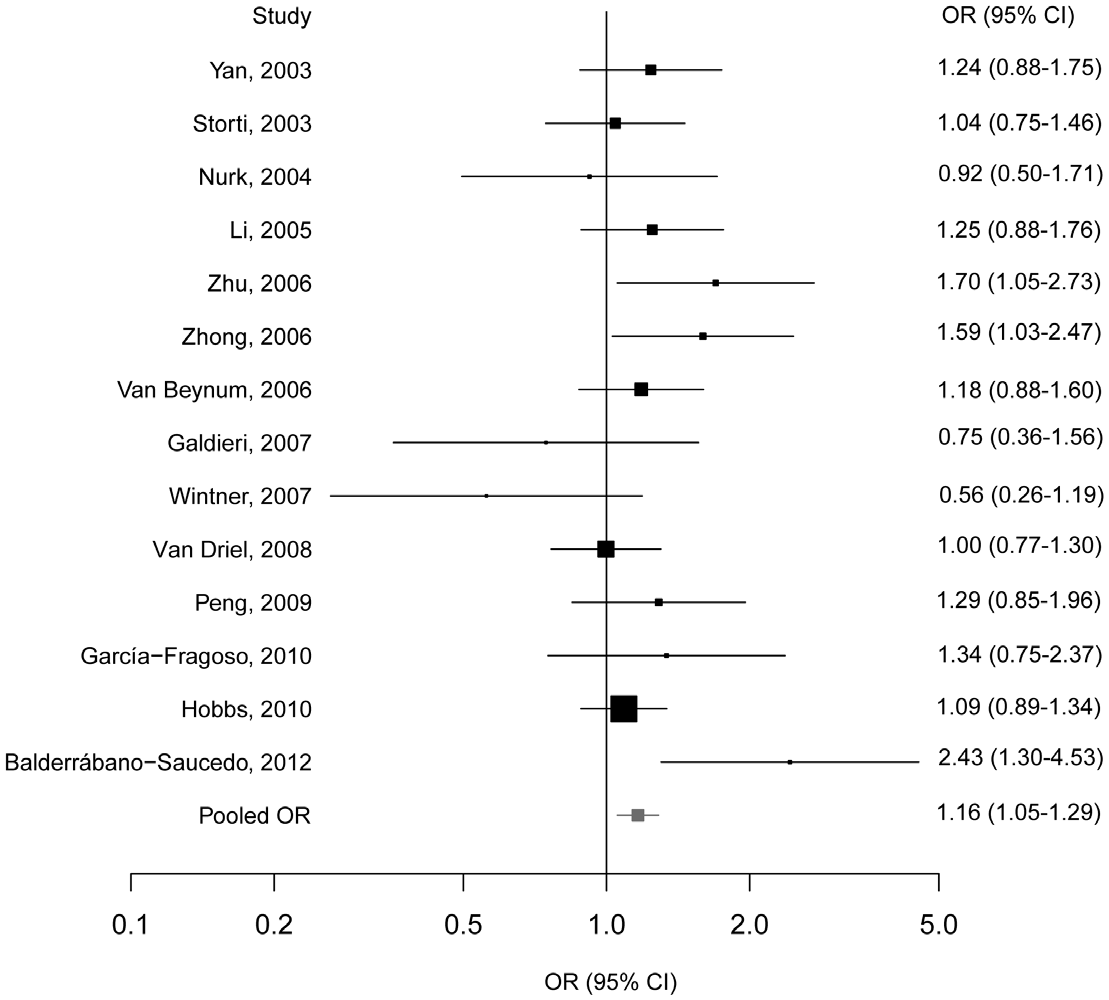
The forest plots of ln(OR) with 95%CIs for the *MTHFR* C667T in mothers for CHDs. Fixed-effects pooled OR = 1.16, 95% CI = 1.05–1.29, *P* = 0.003; *χ* ^2^ = 18.20, *P*_heterogeneity_ = 0.150.

There was no evidence of heterogeneity among 14 studies of the *MTHFR* C677T polymorphism in mothers (*P*_heterogeneity_ = 0.150), thus the fixed-effects model was adopted. Significant association between the variant and CHDs was detected, with the T allele conferring a pooled OR of 1.16 (95% CI = 1.05–1.29) (Figure 3). The sensitivity analysis showed the pooled OR was not dramatically changed by any single study, indicating the robust stability of current results (Table S2). Besides, there was no publication bias found by either the funnel plot (Figure S2) or the Egger’s test (*P*_egger_ = 0.981).

To explore the association of *MTHFR* C677T polymorphism with the risk of CHDs in different populations, stratified analysis by ethnicity was conducted (Table 2). No significant heterogeneity was found in the Asian (*P*_heterogeneity_ = 0.750) or European (*P*_heterogeneity_ = 0.334) population, whereas the heterogeneity appeared in the mixed population (*P*_heterogeneity_ = 0.028). The polymorphism presented significantly increased risk of CHDs in the Asian population (OR = 1.36, 95% CI = 1.14–1.62), but not in the European (OR = 1.03, 95% CI = 0.87–1.22) or mixed (OR = 1.25, 95% CI = 0.72–2.19) population. The data were additionally stratified into larger-sample-size and small-sample-size subgroups (Table 2). In the larger-sample-size subgroup, significant association between *MTHFR* C677T polymorphism and CHDs risk was detected without heterogeneity (*P*_heterogeneity_ = 0.644; OR = 1.14, 95% CI = 1.02–1.27). However, with regard to the small-sample-size subgroup, there was no evidence of association in the random-effects model (*P*_heterogeneity_ = 0.040; OR = 1.21, 95% CI = 0.87–1.68). The pooled OR was not appraised in the case-control and TDT studies separately because of no study with TDT design.

## Discussion

The overall meta-analysis comprising 29 case-control and TDT studies showed significant association between *MTHFR* C677T polymorphism and CHDs risk in children with publication bias, however it became non-significant after we applied the trim-and-fill method to address the problem of publication bias. Nevertheless, positive results were detected after stratified by ethnicity and sample size in all subgroups except the mixed population. With respective to mothers, there was significant association between the polymorphism and CHDs without heterogeneity, whereas the results differed from each subgroup in the stratified analysis of ethnicity and sample size. To the best of our knowledge, this current meta-analysis firstly integrated the case-control and TDT studies to reflect the precision effect of *MTHFR* C677T polymorphism in CHDs risk.

Although the overall meta-analysis demonstrated negative result after adjustment for publication bias, associations between infant *MTHFR* C677T polymorphism and the risk of CHDs were found in all subgroups barring the mixed population, thus we conjectured that the null association might result from the mixed genetic backgrounds. For stratified analysis by ethnicity, the heterogeneity was still detected in all subgroups, suggesting the ethnicity might not be a source of heterogeneity. With respect to the stratified analysis by sample size, heterogeneity disappeared in small-sample-size subgroup but still existed in the other group, which was probably explained by relatively weaker heterogeneity on subjects’ characteristics and genotyping methods across studies with small sample size. For mothers, the *MTHFR* C677T polymorphism presented significantly elevated risk of CHDs without heterogeneity and publication bias. Moreover, the sensitivity analysis further indicated the stability of our results. In the stratified analysis by ethnicity, no obvious heterogeneity in the European and Asian populations was observed, whereas it remained in the mixed population, which was similar with the results in children. However, significant association was merely found in the Asian population, implying there might be different modes of action for this polymorphism across ethnicities. As far as stratified analysis of sample size was concerned, positive results were detected in the large-sample-size subgroup but not in the small-sample-size, implying the null association was probably caused by limited sample size.

The significant association between infant and maternal *MTHFR* C677T polymorphism and CHDs risk observed in current meta-analysis was supported by multiple studies [2], [11], [23], [26], [31]. For example, Li et al. pointed out that *MTHFR* 677TT in children was a risk factor for CHDs [31]. In addition, association was found between the maternal *MTHFR* 677TT and the presence of CHDs in their offspring in the study by Balderrábano-Saucedo et al. [26]. Furthermore, a meta-analysis published in 2012 also arrived at similar results in children [38]. However, some studies reported that the *MTHFR* C677T polymorphism was just associated with a specific phenotype of CHDs [22]–[23], such as coarctation of the aorta and tetralogy of fallot, suggesting that the results of current meta-analysis should be interpreted cautiously. Moreover, significant association between maternal *MTHFR* C677T polymorphism and the risk of CHDs was detected in our study, but not in the meta-analysis by Yin et al. [38], which probably resulted from different genetic models applied and the bigger sample size of current study.

*MTHFR* C677T polymorphism may influence the occurrence of CHDs through homocysteine. As a free amino acid, homocysteine exists in either the reduced (homocysteine, a thiol) or oxidized (homocystine, a disulfide RSSR) form. Thiols can initiate lipid peroxidation, produce hydroxyl radical, and oxidatively cleave proteins. Therefore, regulating the oxidation state of sulfur-containing amino acids is an important strategy for curbing cellular damage [39]. In pregnancy, homocysteine level may reduce to approximately 60% of the normal level, probably because of a reduction of the albumin. However, hyperhomocysteineaemia appears in pregnancy due to a decrease of MTHFR activity [40]. It is known that MTHFR is one of the central regulatory enzymes in the folate metabolism, and it is likely that not only folate deficiency, but also functional polymorphisms in genes associated with the folate-mediated homocysteine pathway, may contribute to the risk of CHDs [35].

Compared with meta-analyses in 2012 [38], a notable strength of our meta-analysis was the application of the method for combining the results from case-control and TDT studies. Given that case-control study is likely to cause a false positive result in the presence of population stratification, integrating case-control and TDT studies can provide a more accurate and comprehensive elevation for the role of genetic polymorphism in disease. Despite the clear strength, some limitation should be acknowledged. Some heterogeneous natures of studies, including different phenotypes of CHDs, mixed populations, diverse female/male ratios and control match conditions probably produce effect on our results. However, we were unable to perform further analysis due to lack of detail data in some studies. In addition, publication bias was detected in overall meta-analysis for children, which affected the reliability of the results to some extent. Nevertheless, the trim-and-fill method was applied to adjust the asymmetry of funnel plot caused by publication bias.

In conclusion, our meta-analysis demonstrated that both infant and maternal *MTHFR* C677T polymorphisms may be significantly associated with the risk of CHDs. But more studies with large sample sizes are required to confirm current findings and explore the interaction between genetic polymorphism, folic acid intake and CHDs risk.

## Supporting Information

Figure S1 **The funnel plots of the** ***MTHFR*** **C667T in children for CHDs.**
(TIFF)

Figure S2 **The funnel plots of the** ***MTHFR*** **C667T in mothers for CHDs.**
(TIFF)

Table S1 **Sensitivity analysis of pooled OR for MTHFR C667T polymorphism in children.**
(DOCX)

Table S2 **Sensitivity analysis of pooled OR for MTHFR C667T polymorphism in mothers.**
(DOCX)

Checklist S1 **PRISMA checklist.**
(DOCX)
