# Supplementary figures and images for: MTHFR C677T Polymorphism and Risk of Congenital Heart Defects: Evidence from 29 Case-Control and TDT Studies
Source: PLoS One. 2013 Mar 11;8(3):e58041. doi: 10.1371/journal.pone.0058041 (PMC3594197; doi:10.1371/journal.pone.0058041)

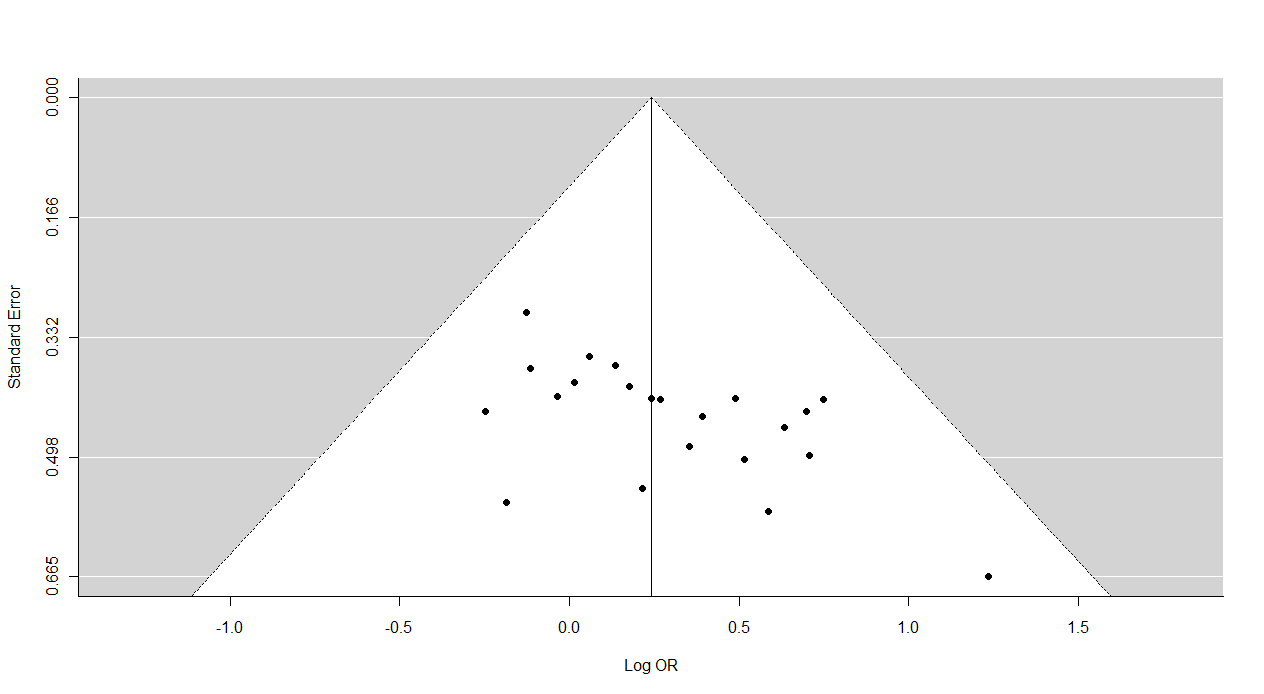

Supplement: Figure S1 — The funnel plots of the MTHFR C667T in children for CHDs. (TIFF) [file pone.0058041.s001.tiff]

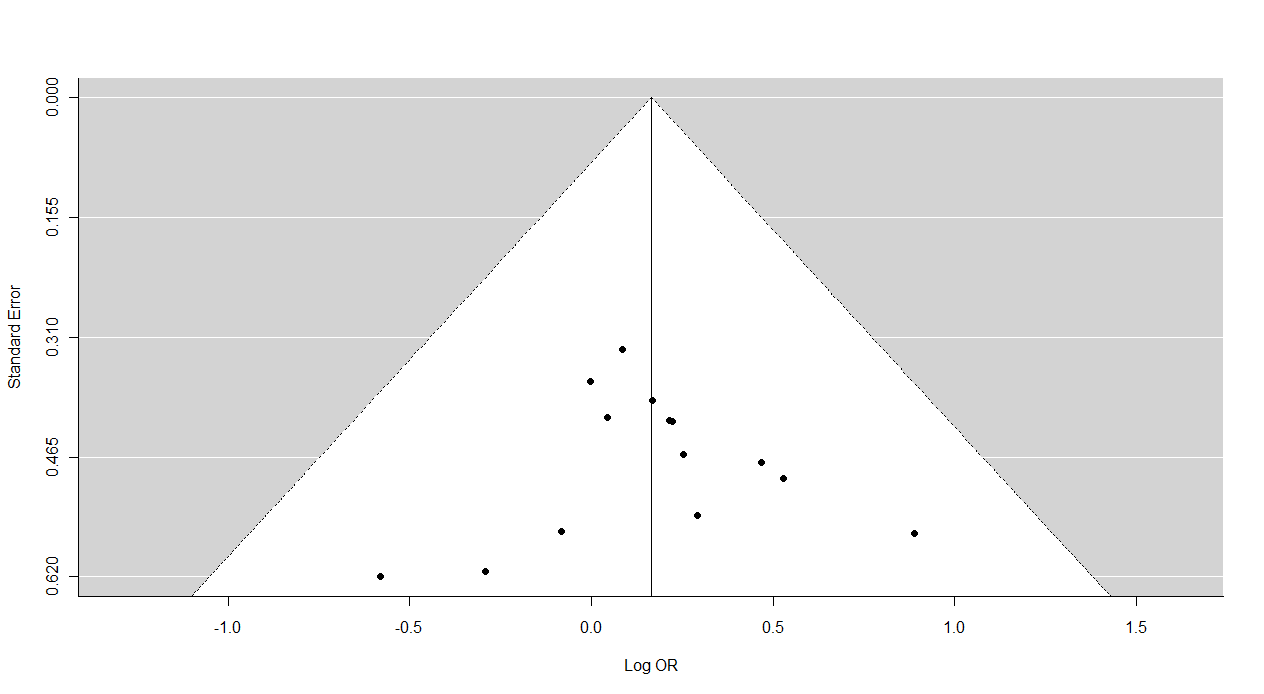

Supplement: Figure S2 — The funnel plots of the MTHFR C667T in mothers for CHDs. (TIFF) [file pone.0058041.s002.tiff]
