# Supplementary material for: MTHFR C677T Polymorphism and Risk of Congenital Heart Defects: Evidence from 29 Case-Control and TDT Studies
Source: PLoS One. 2013 Mar 11;8(3):e58041. doi: 10.1371/journal.pone.0058041 (PMC3594197; doi:10.1371/journal.pone.0058041)
Supplement: Table S1 — Sensitivity analysis of pooled OR for MTHFR C667T polymorphism in children. (DOCX) [file pone.0058041.s003.docx]

**Table S1.** Sensitivity analysis of pooled OR for *MTHFR* C667T polymorphism in children.

| Study omitted | OR (95%CI) | *P* | *P*_heterogeneity_ |
| --- | --- | --- | --- |
| Junker (CC) | 1.28 (1.11-1.48) | 0.001 | 0.000 |
| Wenstrom (CC) | 1.27 (1.11-1.46) | 0.000 | 0.000 |
| Storti (CC) | 1.32 (1.14-1.53) | 0.000 | 0.000 |
| Yan (CC) | 1.30 (1.13-1.51) | 0.000 | 0.000 |
| McBride (TDT) | 1.33 (1.16-1.53) | 0.000 | 0.000 |
| Shaw (CC) | 1.33 (1.15-1.53) | 0.000 | 0.000 |
| Lee (CC) | 1.31 (1.13-1.51) | 0.000 | 0.000 |
| Liu (CC) | 1.29 (1.12-1.49) | 0.000 | 0.000 |
| Li (CC) | 1.30 (1.12-1.50) | 0.000 | 0.000 |
| Zhu (CC) | 1.27 (1.11-1.46) | 0.001 | 0.000 |
| Van Beynum (CC) | 1.32 (1.14-1.53) | 0.000 | 0.000 |
| Hobbs (TDT) | 1.32 (1.14-1.53) | 0.000 | 0.000 |
| Liu (CC) | 1.27 (1.11-1.45) | 0.001 | 0.000 |
| Galdieri (CC) | 1.32 (1.14-1.52) | 0.000 | 0.000 |
| Van Driel (CC) | 1.31 (1.13-1.52) | 0.000 | 0.000 |
| Li (CC) | 1.26 (1.10-1.44) | 0.000 | 0.000 |
| Gong (CC) | 1.30 (1.12-1.50) | 0.000 | 0.000 |
| Marinho (CC) | 1.29 (1.12-1.48) | 0.000 | 0.000 |
| Hu (CC) | 1.29 (1.12-1.48) | 0.000 | 0.000 |
| Kuehl (CC) | 1.28 (1.11-1.47) | 0.001 | 0.000 |
| Xu (CC) | 1.33 (1.16-1.53) | 0.000 | 0.000 |
| García-Fragoso (CC) | 1.30 (1.13-1.50) | 0.000 | 0.000 |

Abbreviations: CC, case-control study; TDT, transmission/disequilibrium test.
