# Supplementary material for: MTHFR C677T Polymorphism and Risk of Congenital Heart Defects: Evidence from 29 Case-Control and TDT Studies
Source: PLoS One. 2013 Mar 11;8(3):e58041. doi: 10.1371/journal.pone.0058041 (PMC3594197; doi:10.1371/journal.pone.0058041)
Supplement: Table S2 — Sensitivity analysis of pooled OR for MTHFR C667T polymorphism in mothers. (DOCX) [file pone.0058041.s004.docx]

**Table S2.** Sensitivity analysis of pooled OR for *MTHFR* C667T polymorphism in mothers.

| Study omitted | OR (95%CI) | *P* | *P*_heterogeneity_ |
| --- | --- | --- | --- |
| Yan (CC) | 1.16 (1.04-1.28) | 0.006 | 0.114 |
| Storti (CC) | 1.18 (1.06-1.31) | 0.002 | 0.123 |
| Nurk (CC) | 1.17 (1.06-1.30) | 0.002 | 0.127 |
| Li (CC) | 1.16 (1.04-1.28) | 0.006 | 0.115 |
| Zhu (CC) | 1.14 (1.03-1.27) | 0.009 | 0.206 |
| Zhong (CC) | 1.14 (1.03-1.27) | 0.009 | 0.186 |
| Van Beynum (CC) | 1.16 (1.05-1.29) | 0.005 | 0.110 |
| Galdieri (CC) | 1.17 (1.06-1.30) | 0.002 | 0.158 |
| Wintner (CC) | 1.18 (1.07-1.30) | 0.001 | 0.270 |
| Van Driel (CC) | 1.19 (1.07-1.33) | 0.001 | 0.162 |
| Peng (CC) | 1.16 (1.05-1.28) | 0.005 | 0.117 |
| García-Fragoso (CC) | 1.16 (1.05-1.28) | 0.004 | 0.117 |
| Hobbs (CC) | 1.19 (1.06-1.33) | 0.003 | 0.126 |
| Balderrábano-Saucedo (CC) | 1.14 (1.03-1.26) | 0.010 | 0.391 |

Abbreviations: CC, case-control study; TDT, transmission/disequilibrium test.
